# Étude descriptive dermoscopique d’une série de 100 carcinome basocellulaires diagnostiqués au Maroc

**DOI:** 10.11604/pamj.2019.34.64.6377

**Published:** 2019-10-01

**Authors:** Meryem Soughi, Mariam Meziane, Salim Gallouj, Fatimazahra Mernissi

**Affiliations:** 1 Service de Dermatologie Vénérologie, Chu Hassan II, Fes, Maroc

**Keywords:** Dermoscopie, carcinome basocellulaire, pigmentation, caractéristiques, phototype, Dermoscopy, basal cell carcinoma, pigmentation, characteristic, prototype

## Abstract

**Introduction:**

La dermatoscopie est une technique d'examen non invasive, permettant de donner un nouveau regard de la morphologie clinique des lésions pigmentées et des tumeurs cutanées. Nous montrons à travers notre série les caractéristiques morphologiques dermotoscopiques du carcinome basocellulaire (CBC) chez notre population.

**Méthodes:**

Notre étude est une étude prospective unicentrique étalée sur une période de 2 ans. Nous avons utilisé le dermoscope chez tous les patients ayant un CBC. L'analyse statistique était réalisée à 'aide du logiciel SPSS version 17.

**Résultats:**

On avait recensé 100 CBC, L'âge moyen des patients était de 51,87 ans, avec un sex ratio F/H = 0,6. Le visage était la localisation la plus fréquente et la plupart des patients étaient de phototype III et IV. On a distingué des critères dermatoscopiques classiques et non classiques. On a montré qu'il existe une relation significative entre le phototype et le degré de la pigmentation des CBC.

**Conclusion:**

Dans ce présent travail, le dermoscope était bénéfique d'une part pour détecter les CBC de petite taille, d'autre part pour faciliter le diagnostic des CBC pigmentés.

## Abstract (en)

**Introduction:**

Dermatoscopy is an innovative non-invasive technique for clinical morphology of pigmented lesions and skin tumor. The purpose of our study was to highlight the morphological and dermoscopic features of basal cell carcinoma (CBC) in our study population.

**Methods:**

We conducted a prospective unicentric study over a period of 2 years. We used dermoscopy in all patients with BCC. Statistical analysis was performed using SPSS software version 17.

**Results:**

The study included 100 patients with BCC, the average age of patients was 51.87 years, with a sex ratio F/M = 0.6. BCC most commonly occurred in the face and most patients were prototype III and IV. We established conventional and non-conventional dermatoscopic criteria. Our study showed that there is a significant relationship between prototype and the degree of BCC pigmentation.

**Conclusion:**

This study highlighted the key role of dermoscopy in detecting small-sized BCC and in facilitating the diagnosis of pigmented BCC.

## Introduction

Le carcinome basocellulaire (CBC) est une tumeur épithéliale maligne à croissance lente, c'est le plus fréquent des tumeurs malignes cutanées [1]. La dermatoscopie aussi appelée microscopie de surface ou sous cutanée ou épiluminescence est une technique d'examen non invasive, permettant de donner un nouveau regard de la morphologie clinique des lésions pigmentées et des tumeurs cutanées [2]. Le point cardinal dermoscopique du diagnostic de CBC est l'absence de réseau pigmenté associé à au moins l'un des six éléments suivants: les nids ovoïdes, l'aspect en roue dentée, l'aspect en feuille d'érable, la vascularisation en tronc d'arbre, les globules gris bleu et la présence d'une ulcération [3,4]. L'objectif de l'étude est de calculer les fréquences des 6 critères de Menzies sur 100 CBC, ainsi que de corréler le phototype cutané des patients avec le caractère pigmenté de cette tumeur.

## Méthodes

Notre étude est une étude prospective unicentrique étalée sur une période de 2 ans du mois 03/10 au mois 03/12, menée au Service de Dermatologie CHU HASSAN II de Fès. Nous avons inclus dans cette étude tous les patients ayant des lésions de carcinome basocellulaire nécessitant un examen dermatologique couplé à un examen dermoscopique. Ces patients provenaient soit de l'activité de consultation du Service de Dermatologie, soit de l'activité d'hospitalisation ou encore de l'activité de gestion des demandes d'avis spécialisés en dermatologie. On a utilisé le dermoscope HEINE DELTA 20, c'est un dermoscope à immersion, possédant une optique à haute résolution avec un système de lentille achromatique, et un grossissement de 10 fois, combiné à un adaptateur pour un appareil photo numérique Canon. Tous les patients ont bénéficié d'un examen clinique et dermoscopique puis des photos ont été prises pour chaque lésion. Les informations étaient recueillies sur une fiche d'exploitation. Les lésions ont bénéficiées d'une exérèse afin d'avoir une confirmation histologique. L'examen clinique, dermoscopique et la prise des photos ont été faites par un seul examinateur. Les données ont été validées et analysées au laboratoire d'épidémiologie, de recherche clinique et santé communautaire de Fès. L'analyse statistique était réalisée à l'aide du logiciel SPSS version 17. Une valeur p<0,05 était considérée comme significative.

## Résultats

**Figure 1:**
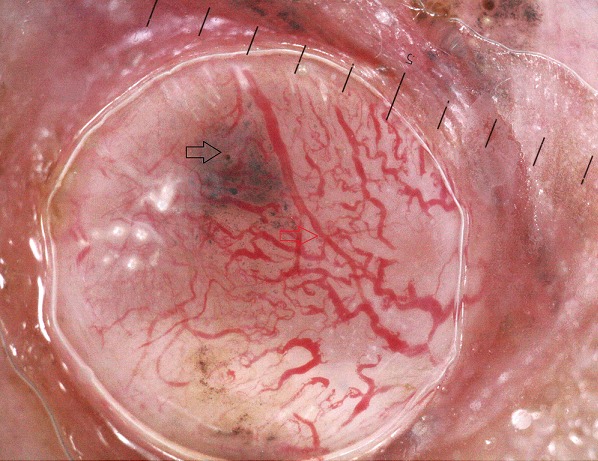
Aspect en tronc d´arbre (flèche rouge) des globules gris bleu (flèche noire) (le pigment occupe moins de 30% de la lésion)

**Figure 2:**
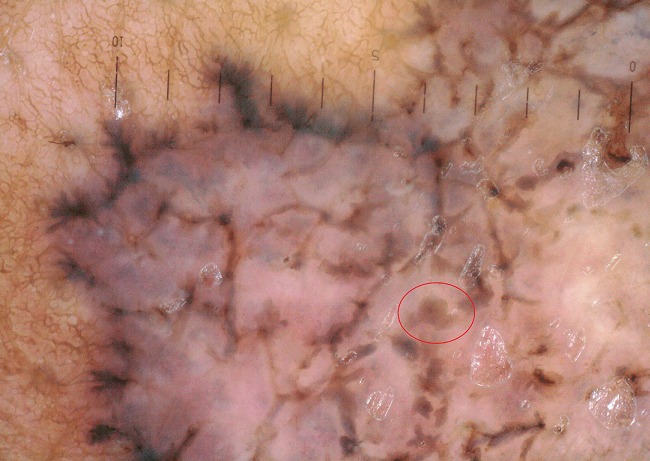
Image dermoscopique d’une carcinome basocellulaire sctructures concentriques (cercle rouge) carcinome basocelulaire pigmenté ( la pigmentation occupe entre 30 et 70% de la lésion)

**Figure 3:**
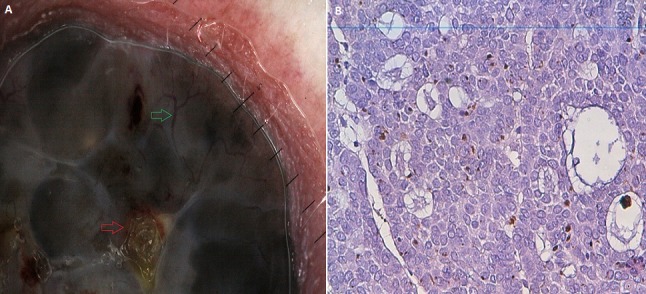
Aspect dermoscopique: présence d´une pigmentation diffuse, ulcération centrale (flèche rouge), aspect en tronc d´arbre ( flèche verte ). La pigmentation occupe plus de 70% de la lésion; (B) aspect histologique: présence du pigment au niveau du derme 40× (collection service d´anatomopathologie CHU HASSAN II FES)

**Figure 4:**
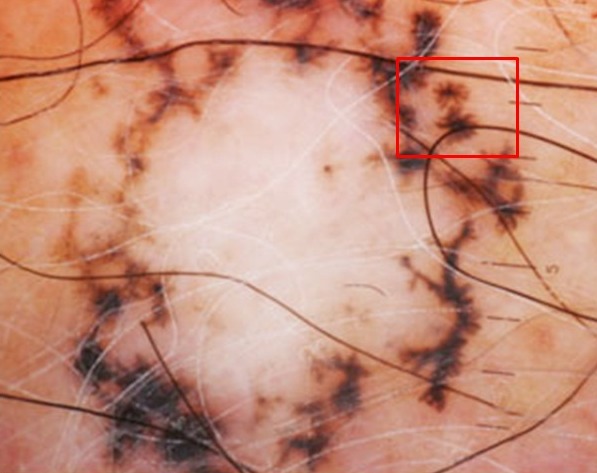
Image dermoscopique d´un carcinome basocellulaire: aspect en roue dentée (carreau rouge)

**Figure 5:**
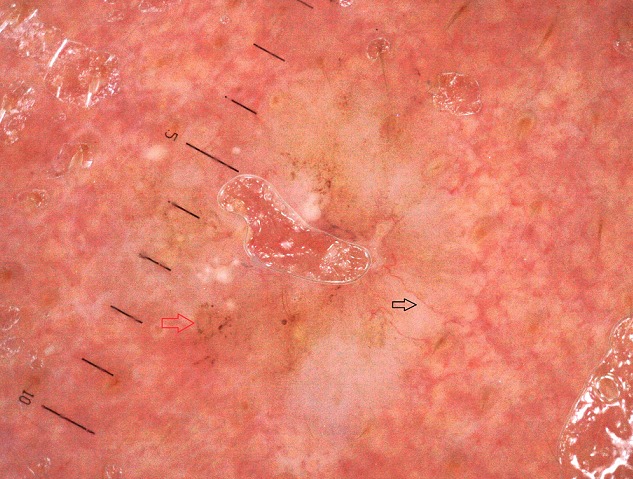
Image dermoscopique d´un CBC: multiples globules gris bleu (flèche rouge), fine télangiectasie (flèche noire)

On a réalisé la dermatoscopie dans 100 carcinomes basocellulaires. Les caractéristiques épidémiologiques: (Tableau 1). Classification des CBC selon la pigmentation dermatoscopique (Tableau 2) on les a classé selon le degré de la pigmentation dermatoscopique en: non pigmenté: caractérisé par l'absence de pigment de couleur brune, noire, grise ou bleue, ce type était observé dans 22% des lésions; légèrement pigmenté: défini par une pigmentation occupant moins de 30% de la lésion, retrouvé dans 29% des lésions (Figure 1); pigmenté: dont la pigmentation occupe entre 30 et 70% de la lésion; il a été décrit dans 16% des lésions (Figure 2); très pigmenté: où la pigmentation occupe plus de 70% de la lésion, il représentait 33% des cas (Figure 3 A,B). Les critères dermoscopiques des CBC (Tableau 3, Tableau 4). On a classé les critères dermatoscopiques du CBC retrouvés en: critères dermatoscopiques dits classiques qui sont: les ulcérations dans 61%, (Figure 3 A,B), les nids ovoides dans 52%,. les télangiectasies ou l'aspect en tronc d'arbre observé dans 47% des lésions (Figure 1) les globules gris bleu dans 38% (Figure 1), les feuilles d'érables (7%) (Figure 2) et les roues dentées dans 3% (Figure 4); des caractéristiques locales ne peuvent être classées dans la catégorie précédente, qu'on a qualifié de critères non classiques qui sont: les points gris bleu dans 19% des lésions (Figure 5), les érosions retrouvées dans 18% des lésions, les fines télangiectasies dans 6% (Figure 5) et les structures concentriques dans 2% (Figure 2); corrélation de la pigmentation des CBC et le phototype (Tableau 5). On a évalué l'association entre le phototype et le degré de la pigmentation des carcinomes basocellulaires: 60,4% patients de phototype II et III avaient un CBC non pigmentés alors que personne de phototype IV et V n'avaient un CBC non pigmenté. Le test chi-2 était très significatif avec une valeur de 0, 000; 39% patients de phototype II et III et 15,3% phototype IV et V avaient un CBC légèrement pigmentés. Le test chi-2 n'était pas significatif avec une valeur de 0,92 ; 31,6% patients de phototype IV et V et 17,4% de phototype II et III avaient un CBC pigmenté. Le test chi-2 n'était pas significatif avec une valeur de 0,23 ; 73,7% patients de phototype IV et V et 26% de phototype II et III avaient un CBC très pigmenté. Le test chi-2 était très significatif avec une valeur de 0, 002.

**Tableau 1:** Les caractéristiques épidémiologiques

Age	51,87 (20-80) ans
sexe	F/H=0,6
Localisation(%)	Visage(76,2), cuir chevelu (20), tronc (2), cou (1)
phototype(%)	III(51,5), IV (36,6), II(5,9), V(5,9)
La forme clinique(%)	Nodulaire(63), superficiel(36), ulcérant (15%), sclérodermiforme (1%), pigmenté (53%)

La forme clinique(%) Nodulaire(63), superficiel(36), ulcérant (15%), sclérodermiforme (1%), pigmenté (53%)

**Tableau 2:** Classification des carcinomes basocellulaires selon la pigmentation dermoscopique

	Clinique(%)	Dermoscopie(%)		
Pigmentation	53	Non pigmenté	22	
		Légérement pigmenté	29	78
		Pigmenté	16	
		Très pigmenté	33	

**Tableau 3:** Les critères dermoscopiques classiques

Les critères dermoscopiques classqiues(%)	
ulcération	61
Nids ovoides	52
télangièctasies	47
Globules gris bleu	38
Feuilles d’érable	7
Roues dentées	3

**Tableau 4:** Les critères dermoscopiques non classiques

Les critères non classiques(%)	
Points gris bleu	19
Erosions	18
Fines télangièctasies	6
Strucutres concentriques	2

**Tableau 5:** Correlation de la pigmentation dermoscopique des carcinomes baso cellulaires avec le phototype des patients

pigmentation				
phototype	Non pigmenté	Légèrement pigmenté	pigmenté	Très pigmenté
I	0			
II	60,4	39	17,4	26
III				
IV	0	15,3	31,6	73,3
V				

## Discussion

Le carcinome basocellulaire (CBC) est une tumeur épithéliale maligne à croissance lente, atteint en général les sujets d'âge moyen de phototype clairs. Sa fréquence est en nette augmentation notamment chez les sujets jeunes, ce qui le témoigne l'âge moyen de nos patients, constituant ainsi un problème de santé publique [5]. Il se présente sous différentes formes cliniques: plan à bordure perlée, nodulaire, superficiel, ulcéreux, sclérodermiforme et pigmenté. L'incidence de la forme pigmentée est de 6,7 à 8,5% en fonction de la race [6]. Le problème majeur, c'est que cette forme est souvent confondue avec les lésions mélanocytaires notamment le mélanome, en raison de leur croissance et leur pigmentation irrégulières. Elle peut également être confondue avec des lésions cutanées bénignes comme les naevus dysplasiques, les naveus de spitz, les kératoses séborrhèiques pigmentées. L'algorithme dermoscopique classique du CBC repose sur l'absence de réseau pigmenté associé à au moins l'un des six éléments suivants: les nids ovoïdes, l'aspect en roue dentée, l'aspect en feuille d'érable, la vascularisation en tronc d'arbre, les globules gris-bleu et la présence d'une ulcération [3]. Dans notre série, les 6 critères classiques ont été retrouvés dans 72% des cas. Mais à des taux variables. On a constaté une fréquence plus élevée pour les nids ovoïdes, l'aspect en tronc d'arbre et une rareté pour l'aspect en roue dentée, qui sont très spécifiques (100%) [7], rejoignant ainsi les données de la littérature [4]. La majorité des caractéristiques positives sont des variantes de la pigmentation confinées à des zones bien circonscrites morphologiquement. Et contrairement au mélanome, l'ulcération est un critère relativement présent au début du CBC. Un autre critère distinctif du mélanome est que Les CBC pigmentés sont moins multicolores que le mélanome, avec seulement 11% ayant cinq ou six couleurs (marqué de l´ocre, brun foncé, noir, gris, bleu et rouge) [4,8]. Par ailleurs, d'autres caractéristiques dermatoscopiques dites non classiques ont également été rapportées, il s'agit essentiellement de petites érosions qui précèdent l'apparition des ulcérations, de fines télangiectasies considérées comme des ébauches pour l'aspect en tronc d'arbre, des structures concentriques comme ébauche de roues dentées, et de multiples points gris bleu [9] ainsi que des structures blanches brillantes [10,11]. Ces éléments ont été observés essentiellement dans les CBC superficiels ce qui permet de détecter les lésions précocement. En effet, dans notre série parmi 100 lésions tumorales suspectes de CBC, 14 lésions ont été découvertes fortuitement à l'examen dermatoscopique et confirmés par l'histologie, parmi lesquels des CBC de très petite taille (Figure 5).

Le CBC peut également présenter des critères dermatoscopiques des lésions mélanocytaires, notamment de multiples points noirs bruns, un voile blanc bleu, une dépigmentation irrégulière, une surface irrégulière, une vascularisation à type de vaisseaux en pointillés, en virgule, des pseudopodes. Mais aussi des critères vus dans les lésions cutanées non mélanocytaires comme les pseudokystes cornées. Ces critères mélanocytaires existent d'autant plus que la lésion est pigmentée et leur fréquence augmente linéairement avec la pigmentation [8,9] (Figure 3 A,B). Dans notre travail l'aspect mélanocytaire le plus souvent retrouvé était le voile blanc bleu détecté dans 17% des lésions. Nos résultats ont montrés que la dermatoscopie a permis de révéler 25% des CBC non pigmentés cliniquement, contre 30% par rapport à une étude multicentrique [12]. Par contre on a noté un nombre élevé de CBC pigmenté par rapport à la literature. La detection de la pigmentation est utile pour sélectionner les bons répondeurs au traitement par la photothérapie dynamique, puis que la detection d'une pigmentation non visible cliniquement constitue un facteur de mauvaise réponse [13]. Une étude multicentrique faite en Italie, Australie, et l'Allemagne, avait noté 9% des CBC très pigmentés [9] alors que dans notre série, le CBC très pigmenté était le type le plus retrouvé dans 38,6% des lésions (Figure 3 A,B), ceci pourrait être lié au phototype des patients qui sont majoritairement de type III et IV, quoique ce critère n'est à lui seul suffisant. Pour confirmer cette hypothèse, on a fait une corrélation entre le phototype et la pigmentation du CBC et on a constaté que les CBC non pigmentés se voient exclusivement chez les patients de phototype clair alors que les très pigmentés se voient essentiellement chez les phototypes foncés et qu'il existe une relation. La forme très pigmentée constitue donc la forme la plus difficile à distinguer des naevus et des mélanomes ce qui suggère que l'interprétation dermatoscopique des CBC doit toujours évaluer l'aspect global de la lésion en recherchant les patrons classiques du CBC, les caractéristiques dermatoscopiques locales qui sont les patrons non classiques tout en prenant en compte le phototype du patient. Dans notre contexte, l'utilisation de la dermatoscopie comme moyen pour faire la part entre un carcinome basocellulaire pigmenté, un mélanome ou bien une autre tumeur pigmentée était très intéressante car elle permet d'orienter la démarche thérapeutique.

**Limites:** au cours de notre étude prospective, nous avons été limités par le faible nombre de malade. La présence d'un seul examinateur La plupart des patients consultent tardivement à des stades tumoraux limitant l'apport et l'utilisation de l'examen dermatoscopique. Il n'existe pas d'étude à l'échelle nationale permettant la comparaison de nos résultats. Les patients examinés sont dans la majorité des cas de phototype foncé ce qui explique que l'interprétation et l'analyse dermatoscopiques étaient délicates.

## Conclusion

Dans ce présent travail, le dermoscope était bénéfique d'une part pour détecter les CBC de petite taille. D'autre part pour faciliter le diagnostic des CBC pigmentées, constituant la forme la plus fréquente dans notre contexte où domine le phototype foncé, et dont l'interprétation est difficile et posent un véritable diagnostic différentiel avec le mélanome.

### État des connaissances actuelles sur le sujet

- Le carcinome basocellulaire est une tumeur cutanée atteint en général les sujets d'âge moyen de phototype clairs;
- Il existe 6 critères dermoscopiques classiques pour le diagnostic de carcinome basocelllaire mais aussi des critères non classiques;
- L'incidence de la forme pigmentée est de 6,7 à 8,5% en fonction de la race.

### Contribution de notre étude à la connaissance

- On a constaté que les critères dermoscopiques non classiques sont essentiellement vus dans les carcinomes basocellulaires très superficiels ce qui a permis de détecter les lésions précocement;
- On a trouvé une incidence élevée des carcinomes basocellulaires pigmentés dans notre série par rapport à la literature;
- Notre étude a confirmé qu'il existe une relation significative entre le phototype et le degré de la pigmentation des CBC.

## Conflits d’intérêts

Les auteurs ne déclarent aucun conflit d’intérêts.
